# Anatomical and Molecular Properties of Long Descending Propriospinal Neurons in Mice

**DOI:** 10.3389/fnana.2017.00005

**Published:** 2017-02-06

**Authors:** Jamie R. Flynn, Victoria L. Conn, Kieran A. Boyle, David I. Hughes, Masahiko Watanabe, Tomoko Velasquez, Martyn D. Goulding, Robert J. Callister, Brett A. Graham

**Affiliations:** ^1^School of Biomedical Sciences and Pharmacy, University of NewcastleCallaghan, NSW, Australia; ^2^Hunter Medical Research InstituteNewcastle, NSW, Australia; ^3^Institute of Neuroscience and Psychology, University of GlasgowGlasgow, UK; ^4^Department of Anatomy, Hokkaido University School of MedicineSapporo, Japan; ^5^Molecular Neurobiology Laboratory, The Salk Institute for Biological StudiesLa Jolla, CA, USA

**Keywords:** propriospinal, neuroanatomy, developmental genetics, inhibitory, morphology, synapse

## Abstract

Long descending propriospinal neurons (LDPNs) are interneurons that form direct connections between cervical and lumbar spinal circuits. LDPNs are involved in interlimb coordination and are important mediators of functional recovery after spinal cord injury (SCI). Much of what we know about LDPNs comes from a range of species, however, the increased use of transgenic mouse lines to better define neuronal populations calls for a more complete characterisation of LDPNs in mice. In this study, we examined the cell body location, inhibitory neurotransmitter phenotype, developmental provenance, morphology and synaptic inputs of mouse LDPNs throughout the cervical and upper thoracic spinal cord. LDPNs were retrogradely labelled from the lumbar spinal cord to map cell body locations throughout the cervical and upper thoracic segments. Ipsilateral LDPNs were distributed throughout the dorsal, intermediate and ventral grey matter as well as the lateral spinal nucleus and lateral cervical nucleus. In contrast, contralateral LDPNs were more densely concentrated in the ventromedial grey matter. Retrograde labelling in *GlyT2*^GFP^ and *GAD67*^GFP^ mice showed the majority of inhibitory LDPNs project either ipsilaterally or adjacent to the midline. Additionally, we used several transgenic mouse lines to define the developmental provenance of LDPNs and found that V2b positive neurons form a subset of ipsilaterally projecting LDPNs. Finally, a population of Neurobiotin (NB) labelled LDPNs were assessed in detail to examine morphology and plot the spatial distribution of contacts from a variety of neurochemically distinct axon terminals. These results provide important baseline data in mice for future work on their role in locomotion and recovery from SCI.

## Introduction

The propriospinal system is comprised of spinal interneurons with longitudinal axonal projections that extend outside their segment of origin, forming a network that connects motor and sensory circuits throughout the length of the spinal cord (propriospinal neurons; PNs). This intraspinal network is important for the propagation of supraspinal signals (Cowley et al., 2008, 2010), interlimb coordination (Lloyd and McIntyre, 1948; Miller et al., 1973; Ballion et al., 2001; Juvin et al., 2005, 2012; Zaporozhets et al., 2006; Pocratsky et al., 2014), sensorimotor integration (see Alstermark and Isa, 2012), and functional recovery from spinal cord injury (SCI) via collateral sprouting and the formation of “detour” circuits (Bareyre et al., 2004; Vavrek et al., 2006; Courtine et al., 2008; Fenrich and Rose, 2009; Flynn et al., 2011; Filli et al., 2014; Benthall et al., 2017).

Classic studies by Sir Charles Sherrington (Sherrington and Laslett, 1902, 1903), Hans Kuypers (Sterling and Kuypers, 1968; Giovanelli Barilari and Kuypers, 1969; Rustioni et al., 1971; Molenaar and Kuypers, 1978) and János Szentágothai (Szentagothai, 1951, 1964) identified multiple PN subtypes as defined by their cell body locations and projection patterns (i.e., long, short, ascending, descending, commissural and so on; see Flynn et al., 2011). More recent work has focused on the neurochemical phenotype and transcription-factor expression within various PN subpopulations (Liu et al., 2010; Brockett et al., 2013; Ni et al., 2014). Recent advances in viral targeting, transgenic mouse lines and optogenetics has allowed the molecular profile of PNs and the circuits they form to be studied in great detail. Of note, it has become increasingly important to define PNs by their developmental origin, which is based on early transcription factor expression (Goulding, 2009). These developmentally defined interneuron populations (i.e., V0-V3 and dI0-dI6) form anatomically and functionally discrete spinal neuron classes that are amenable to genetic targeting. For example, a subset of cervical premotor PNs were identified as derivatives of the V2a (Chx10 positive) interneuron population and subsequent ablation revealed they play a critical role in forelimb reaching (Azim et al., 2014). Similarly, V2a interneurons also form a group of thoracic, premotor PNs that project to the lumbar cord to innervate ankle dorsiflexor muscles (Ni et al., 2014). To our knowledge, the contribution of other developmentally defined interneuron classes has not been investigated across PN populations.

Another subset of PNs, termed “long descending” PNs (LDPNs), is currently the subject of a similar anatomical and molecular investigation (Reed et al., 2006; Brockett et al., 2013; Ni et al., 2014). LDPNs are located in the cervical and upper thoracic spinal cord and their axons project to the lumbar enlargement (Reed et al., 2006; Conta and Stelzner, 2009; Cowley et al., 2010; Brockett et al., 2013). This anatomical arrangement, when considered in conjunction with early work on their functional properties (Lloyd and McIntyre, 1948; Vasilenko, 1975; Alstermark et al., 1987b), suggest they are important mediators of interlimb coordination. More recently, LDPNs have been highlighted as important components in the “re-wiring” of descending motor projections following SCI. Specifically, they make detour circuits for descending supraspinal axons to bypass lesion sites and innervate spinal segments below the injury (Bareyre et al., 2004; Vavrek et al., 2006). LDPNs are particularly suited for these recovery roles as their axons project over long distances in the spinal cord, and are quite resilient to cell death following axonal damage (Conta and Stelzner, 2004; Conta Steencken and Stelzner, 2010; Siebert et al., 2010).

Given the critical role of LDPNs in locomotor pattern generation, coordination between fore- and hind-limbs and their capacity to promote functional recovery after SCI, a better understanding of their morphological, physiological, neurochemical and developmental features remains a high priority. Additionally, the increased use of transgenic mouse lines to better define neuronal populations calls for detailed characterisation of LDPNs in mice. Therefore, the aim of this study was to provide a survey of this functionally important population by defining the anatomical, morphological and molecular features of LDPNs in mice.

## Materials and Methods

### Animals

All experimental procedures were approved by either the Salk Animal Care and Use Committee or the University of Newcastle Animal Care and Ethics Committee, and carried out in accordance with the National Institutes of Health and the National Health and Medical Research Council guidelines. Retrograde tracing experiments using Fluorogold were performed in wildtype ICR and transgenic *GlyT2*^GFP^, *GAD67*^GFP^, *En1*^Cre^;*Thy1*^floxstop−YFP^, *Gata3*^Cre^;*Thy1*^floxstop−YFP^, and *Sim1*^Cre^;*Thy1*^floxstop−YFP^ mice (see Table 1 for details). The ICR strain was used as all transgenic mice were generated using 129S1/SvImj and C57BL6 lines and maintained on an ICR mouse background. First generation offspring from these crosses were used in this study. Additional tracing experiments used DiI to allow Neurobiotin (NB) filling of LDPNs during electrophysiology in spinal cord slices and subsequent morphological analysis. This work was performed in C57BL6 mice to allow direct comparison with electrophysiological data, typically collected in the C57BL6 line. Importantly, there was no appreciable difference in labelling pattern between ICR and C57BL6 mice.

**Table 1:** **Genetic mouse strain details**.

Strain	Age	Number	Source/Ref
WT (ICR)	P29–30	5	-
WT (C57BL6)	P22–29	9	-
GAD67^GFP^	P31	3	Tamamaki et al. (2003)
*GlyT2*^GFP^	P32	3	Zeilhofer et al. (2005)
*En1*^Cre^;*Thy1*^floxstop−YFP^ (V1)	P25	2	Sapir et al. (2004)
*Gata3*^Cre^;*Thy1*^floxstop−YFP^ (V2b)	P26 and P47	2	Zhang et al. (2014)
*Sim1*^Cre^;*Thy1*^floxstop−YFP^ (V3)	P25	2	Zhang et al. (2008)

### Fluorogold Tracing Injections

**Figure 1:**
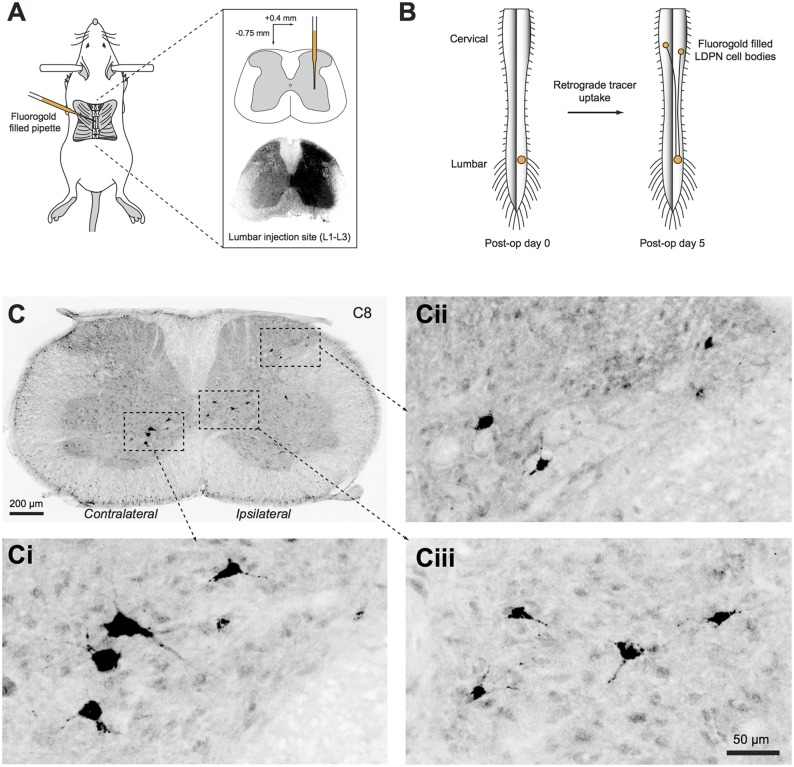
**Unilateral injection of retrograde tracer into the right lumbar spinal cord. (A)** Schematic summarising the surgical approach to retrogradely label long descending propriospinal neurons (LDPNs). *Inset upper*: pipette tip positioned in intermediate zone. *Inset lower*: Typical spread of Fluorogold after L2 injection. **(B)** Mice recovered for 5 days after surgery to allow retrograde transport of Fluorogold from the injection site in the lumbar cord to both ipsilateral and contralateral LDPN cell bodies in cervical spinal segments. **(C)** Cervical spinal cord section immunostained for Fluorogold 5 days after injection. **(Ci–iii)** High power insets showing LDPNs in ipsilateral and contralateral regions of the cervical grey matter (as denoted by dashed boxes in **C**).

Wildtype (ICR) and transgenic mice were anaesthetised with isoflurane (5% induction, 1%–3% maintenance) and a T12 laminectomy was performed to expose the L2 spinal segment. The overlying dura mater was cleared and an injection pipette filled with 4% (w/v) Fluorogold dissolved in normal saline (Fluorochrome, CO, USA) was advanced to a depth of 0.75 mm from the surface of the spinal cord, 0.4 mm lateral to the midline (Figure 1A). Fluorogold was injected (20–50 nL) with pressure pulses using a picospritzer (20 psi, 5 ms duration). The pipette was left in place for 5 min and withdrawn over another 5 min to minimise Fluorogold spread along the injection tract. Animals with injection sites that extended contralaterally (i.e., into the grey matter on the left site of the spinal cord; determined after sectioning) or did not cover the entire dorsal horn, intermediate zone and ventral horn of the right side grey matter were excluded from further analysis. The wound was closed and buprenorphine (0.05–0.1 mg/kg, s.c.) was administered to minimise postoperative discomfort. Animals were fixed by transcardial perfusion of paraformaldehyde (4% in phosphate buffered saline (PBS)) 5 days after injection (predetermined as the optimal survival time for retrograde transport of Fluorogold to label LDPN cell bodies in the cervical cord; Figure 1B). The spinal cord was removed and post-fixed in 4% paraformaldehyde for an additional hour before cryoprotection in 30% sucrose in PBS overnight at 4°C.

### Fluorogold Labelling and Analysis

Cervical and upper thoracic segments were sectioned at 40 μm in the transverse plane on a freezing sledge-microtome. Every fifth section was processed with anti-Fluorogold antibody to reveal retrograde-labelled LDPNs, and tissues expressing transgenic GFP/YFP were additionally treated with anti-GFP antibody (see Table 2 for details). This was followed by incubation in species-specific secondary antibodies conjugated to Alexa 647 or Alexa 488 (1:500 for both; Molecular Probes, Eugene, OR, USA). All antibodies were made up in 1% normal donkey serum in PBS with 0.3% Triton X-100 (PBST), and all sections were mounted on glass slides in Aqua-Poly/Mount (Polysciences; Warminster, PA, USA). Photomicrographs were captured on an Olympus BX61 microscope (Olympus; Tokyo, Japan) using Metamorph software (Molecular Devices; Sunnyvale, CA, USA). For each section, several images were acquired and digitally stitched using Metamorph software (Figure 1C). Injection sites were verified in 60 μm thick transverse sections of the upper lumbar enlargement revealing the endogenous fluorescence of the Fluorogold bolus (Figure 1A).

**Table 2:** **Primary antibodies**.

Antigen	Host	Dilution	Cat. No.	Source/Reference
Fluorogold	Rabbit	1:10000	AB153	Millipore; Temecula, CA, USA
GFP	Chicken	1:500	GFP-1020	Aves Labs; Tigard, OR, USA
VGAT	Goat	1:1000	-	Frontier Institute Co Ltd.; Hokkaido, Japan/Sardella et al. (2011)
VGLUT1	Rabbit	1:5000	135 303	Synaptic systems; Göttingen, Germany.
Parvalbumin	Guinea pig	1:500	PV-GP-Af1000	Frontier Institute Co Ltd.; Hokkaido, Japan.

To map the location of Fluorogold labelled LDPNs in wild-type (ICR) and transgenic mice, photomicrographs of immunostained sections between C2 to T3 were imported into Adobe Illustrator CS4 (Adobe, San Jose, CA, USA) and superimposed on transverse spinal cord slice templates (adapted from Watson et al., 2009). Images were carefully matched to the appropriate cervical or upper thoracic template using the dorso-ventral extent of the dorsal columns and the shape of the ventral horns. The images were rotated and/or uniformly resized to fit the template. LDPN cell body locations were then plotted on the template. The position of LDPN cell bodies within the spinal cord templates was used to determine the lamina of origin (Graham et al., 2008). For transgenic mice, the position of LDPNs that colocalised with GFP or YFP positive neurons was also recorded.

Heat maps were generated to compare the relative distribution and density of LDPN cell bodies throughout the cervical and upper thoracic spinal cord. Spinal cord templates, segmented into grids (15 μm × 15 μm squares), were matched to each section containing LDPN cell bodies. Each square within the grid was scored based on the number of LDPN cell bodies it contained. The individual squares within each spinal segment grid were then coloured based on their score using a conditional formatting function (i.e., ranging from white for 0 to dark red for a maximum of nine LDPN cell bodies). The colour-coded grids were overlaid onto the original spinal cord templates in Adobe Illustrator.

### Neurobiotin Fills

First, LDPNs were retrogradely labelled in wild-type (C57BL6) mice with 2.5% (w/v) DiI dissolved in DMSO using the same surgical procedure described above for Fluorogold injections. DiI was used instead of Fluorogold to enable easier identification of LDPNs as it has brighter fluorescence in thick spinal cord slices. Targeted whole-cell patch-clamp NB fills were carried out in fresh transverse spinal cord slices (250 μm thick) that were maintained in oxygenated artificial cerebrospinal fluid (Graham et al., 2011). After establishing the whole-cell configuration, DiI retrograde-labelled LDPNs were filled via a patch pipette containing 0.2% (w/v) NB (Vector Labs, Burlingame, CA, USA) dissolved in a CsCl-based internal solution (Graham et al., 2011) for 20–30 mins. A comparison group of unlabelled spinal neurons neighbouring retrogradely-filled LDPNs in the same region (termed our “control” group) were also filled with NB for morphological comparison. LDPNs and control neurons were both sampled from spinal segments C4 to T2 and were located in the ventromedial grey matter (laminae VII and VIII), contralateral to the lumbar DiI injection site. Slices containing NB-filled neurons were then immersion-fixed in 4% paraformaldehyde overnight at 4°C, and cryoprotected in 30% sucrose in PBS for subsequent anatomical studies.

### Synaptic Terminal Labelling

Spinal cord slices containing NB-filled neurons were first incubated in Avidin conjugated to rhodamine (diluted 1:1000; Jackson ImmunoResearch, West Grove, PA, USA) to label NB-filled neurons. The entire somatodendritic arbour of recovered neurons was then scanned on a Bio-Rad Radiance 2100 confocal microscope (Hemel; Hempstead, UK) with a krypton–argon laser at 20× magnification. This laser exposure during this initial scanning step potentially quenched some of the fluorescence. Therefore, maximal labelling for subsequent high-power scanning was achieved by re-sectioning slices at 60 μm and re-incubating these sections in Avidin-rhodamine to increase label penetration and replenish the potentially quenched flurophore in LDPN processes. To label putative synaptic inputs onto the filled neurons, selected sections containing the rhodamine-labelled neurons were incubated in a cocktail of primary antibodies against vesicular GABA transporter (VGAT), vesicular glutamate transporter type 1 (VGLUT1) and parvalbumin (PV) for 48 h at 4°C (see Table 2 for details). Sections were then incubated in species-specific secondary antibodies conjugated to Pacific Blue (1:200; Jackson ImmunoResearch, West Grove, PA, USA), Alexa 488 (1:500; Molecular Probes, Eugene, OR, USA), or DyLight 647 (1:500; Jackson ImmunoResearch, West Grove, PA, USA) and mounted on glass slides in Vectashield (Vector Laboratories; Peterborough, UK). Tiled scans to visualise appositions from putative VGAT-, VGLUT1- and PV-expressing axon terminals onto the NB-filled neurons were acquired on a Zeiss LSM710 confocal microscope with Argon multi-line, 405 nm diode, 561 nm solid state and 633 nm HeNe lasers, at 40× magnification with a 1 μm z-separation. Precautions were taken while setting scan parameters to avoid potential bleed-through between fluorochomes.

Overlapping tiled scans of each labelled cell were stitched together to montage the entire somato-dendritic arborisation of each LDPN cell, using the original 20× reconstructions as a template. The morphology of each NB-filled neuron was first reconstructed in 3-dimensions from these overlapping confocal image stacks using Neurolucida for Confocal software (MicroBrightField, Colchester, VT, USA). The precise location of direct appositions from four types of axon terminals were then plotted on the reconstructed neurons, based on their relative expression patterns for VGAT, VGLUT1 and PV, and assigned to one of four potential synaptic input populations. Terminals expressing VGAT were considered segmental inhibitory interneurons (Yasaka et al., 2010), whereas those expressing both VGAT and PV were described as having arisen from premotor interneurons (Alvarez et al., 2005). Terminals that expressed only VGLUT1 were assigned as myelinated afferent fibres and/or corticospinal inputs (Todd et al., 2003; Du Beau et al., 2012), whereas those expressing both VGLUT1 and PV were classified as proprioceptive afferents (Zhang et al., 1990; Clowry et al., 1997).

Total dendritic lengths and branching patterns were measured and compared across LDPNs and control neurons using Neurolucida Explorer 10 software (MicroBrightField, Colchester, VT, USA). A Sholl analysis, also carried out using Neurolucida Explorer 10 software, was performed to determine the number of terminals expressing VGAT, VGLUT1 and PV that apposed the NB labelled neurons on different segments of their somatodendritic arbour. The first shell was set at 10 μm and any contacts found within this were deemed to be on the cell body. All subsequent shells were spaced at 20 μm intervals.

### Statistical Analysis

Group comparisons were made with an unpaired Student’s *t*-test when data sets were normally distributed (assessed by the Kolmogorov-Smirnov test) and had equal variance (SD of both groups differed by less than a factor of 2). In cases where data sets were normally distributed but had unequal variances, Welch’s *t*-test was applied. Non-normally distributed data sets were compared with the Mann-Whitney *U* test. All statistical analysis was performed with GraphPad Prism 6 software (GraphPad Software, La Jolla, CA, USA). Statistical significance was set at *p* < 0.05. Values are presented as mean ± SEM unless otherwise noted.

## Results

The cell body locations of lumbar projecting LDPNs were mapped throughout the cervical and upper thoracic spinal cord in wild-type (ICR) mice. Inhibitory LDPNs were mapped in *GlyT2*^GFP^ (glycinergic) and *GAD67*^GFP^ (GABAergic) mice. LDPNs that express markers of developmentally defined interneurons were quantified in *En1*^Cre^;*Thy1*^floxstop−YFP^ (V1), *Gata3*^Cre^;*Thy1*^floxstop−YFP^ (V2b) and *Sim1*^Cre^;*Thy1*^floxstop−YFP^ (V3).

### Injection Sites

Injection sites were located within the L2 spinal segment (right side of cord) and confined within the borders of the grey matter (Figure 1A). An injection was deemed successful (and appropriate for further study) if Fluorogold spread throughout the ipsilateral dorsal horn, intermediate zone and ventral horn. Approximately 20% of the injections failed to meet these criteria and were excluded. In some cases, the tracer bolus extended rostrocaudally into the L1 or L3 segments, but remained ipsilateral in all cases studied.

### LDPN Cell Body Location

**Figure 2:**
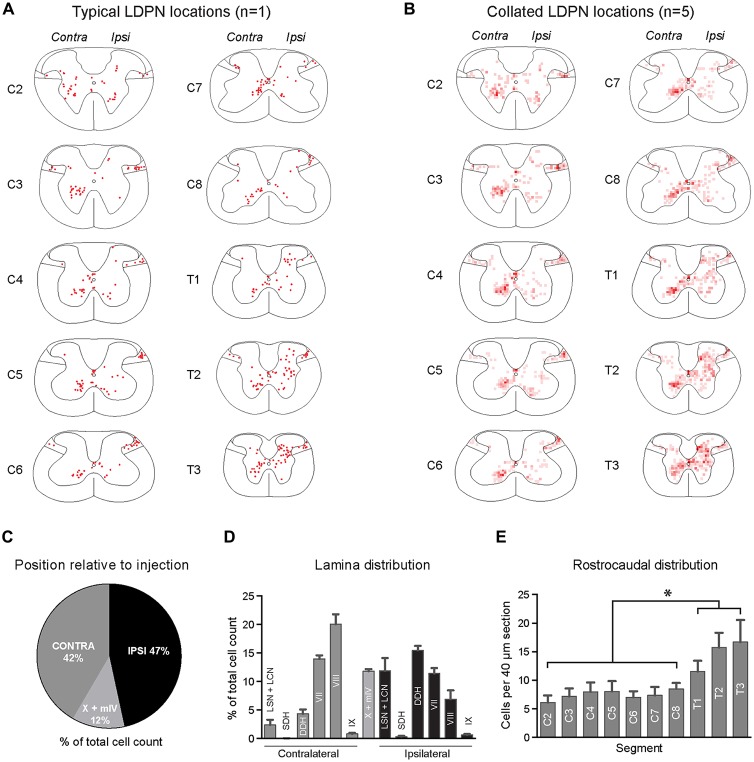
**Lamina distribution of LDPN cell bodies throughout the cervical and upper thoracic spinal cord in wild-type (ICR) mice. (A)** Distribution of LDPN cell bodies in the cervical and upper thoracic spinal cord from one representative animal. **(B)** Heat maps were generated by collating LDPN cell body locations from all wildtype (ICR) mice (*n* = 5). Dark red colouring denotes high LDPN density. **(C)** Incidence of LDPNs across the contralateral, ipsilateral and midline regions. **(D)** Lamina distribution of LDPNs. Ipsilateral LDPNs were prevalent in the deep dorsal horn (IV to VI) and lamina VII and VIII. A large population of ipsilateral LDPNs was also identified in the LSN and LCN. A population of midline LDPNs was located in lamina IV and X. Contralateral LDPNs were clustered in medial lamina VII and VIII. **(E)** The rostrocaudal distribution of LDPNs. The number of LDPNs per 40 μm section increased in more caudal segments. Graphs are comprised of data from five wildtype (ICR) mice. *Denotes *p* < 0.05.

The cell bodies of lumbar projecting LDPNs were mapped between C2 and T3 spinal segments. Retrogradely-labelled LDPNs were found both ipsilateral and contralateral to the lumbar injection site (Figures 2A,B). A similar percentage of LDPNs were found on either side of the cord (46.6 ± 0.8% ipsilateral, 41.6 ± 1.1% contralateral), with the remainder (11.8 ± 0.4%) located close the midline (lamina X and medial lamina IV; Figure 2C).

Ipsilateral LDPNs were distributed widely throughout the grey matter, including the deep dorsal horn (laminae IV to VI; 15.5 ± 0.8%), lamina VII (11.4 ± 0.9%) and lamina VIII (7.0 ± 1.5%). Retrogradely-labelled LDPNs were also found in the ipsilateral lateral spinal nucleus and lateral cervical nucleus (LSN/LCN; 11.9 ± 2.3%; Figures 2A,B,D). Contralateral LDPN cell bodies were numerous around the ventromedial border of laminae VII (14.0 ± 0.6%) and VIII (20.0 ± 1.8%). A population of LDPNs was also identified in the LSN and LCN, however, these were less numerous compared to the ipsilateral side (2.4 ± 0.9% vs. 11.9 ± 2.3%; Figures 2A,B,D). Midline LDPNs were located dorsal to the central canal (Figures 2A,B).

LDPN cell bodies were uniformly distributed throughout the rostro-caudal extent of the cervical cord, but more prevalent in thoracic segments (7.4 ± 0.3 vs. 14.6 ± 1.6 neurons per 40 μm section; *p* = 0.02; Figure 2E).

### Inhibitory LDPN Cell Body Location

**Figure 3:**
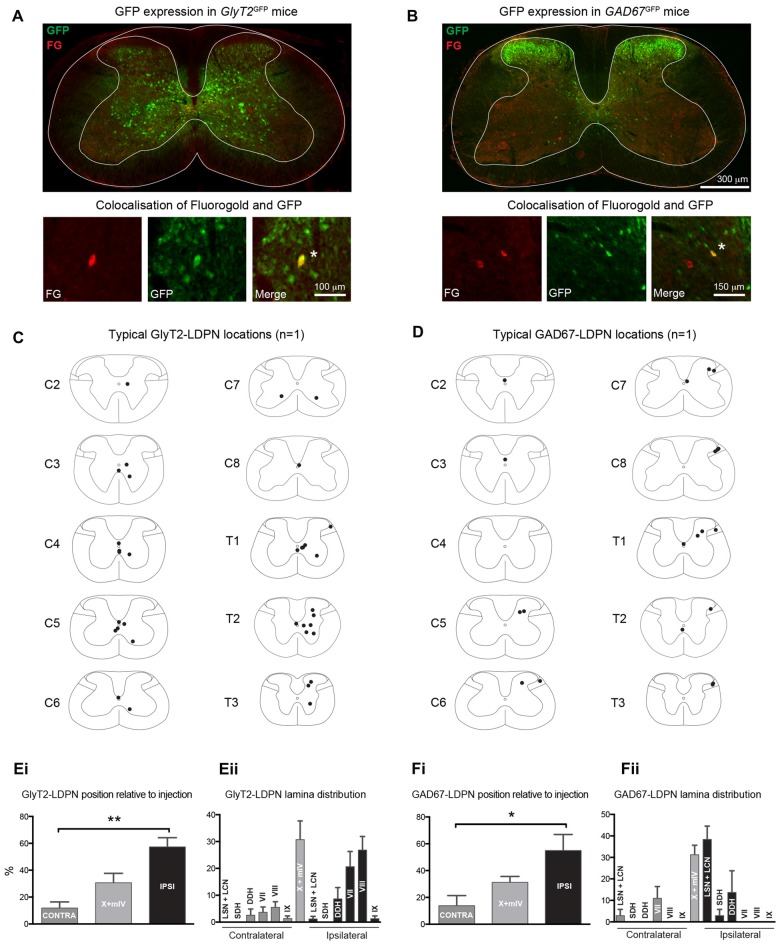
**Identification and location of inhibitory LDPNs. (A,B)** *Upper panels*: Representative immunostained cervical spinal cord sections from *GlyT2*^GFP^ and *GAD67*^GFP^ mice retrogradely-labelled with Fluorogold. Note the differential localisation of glycinergic and GABAergic neurons. *Lower panels*: High magnification images show examples of inhibitory LDPNs identified by Fluorogold and GFP colocalisation (white asterisk). Note, upper and lower panels are taken from separate spinal cord sections. Example distribution of GlyT2-LDPNs **(C)** and GAD67-LDPNs **(D)** neurons, each complied from one representative animal. GlyT2-LDPNs and GAD67-LDPNs were predominantly located either ipsilateral to the lumbar injection site or in the midline of the spinal cord (**Ei,Fi**, respectively). Significantly higher numbers of GlyT2-LDPNs and GAD67-LDPNs were found ipsilateral to the injection site. GlyT2-LDPNs were typically located in the midline (lamina IV and X) and ipsilateral lamina VII and VIII **(Eii)**, while GAD67-LDPNs were mostly found in the midline and the ipsilateral LSN and LCN **(Fii)**. Graphs comprised of data from 3 animals per group. *Denotes *p* < 0.05, **Denotes *p* < 0.005.

The cell body location and incidence of inhibitory LDPNs was assessed via colocalisation of Fluorogold and GFP in *GlyT2*^GFP^ and *GAD67*^GFP^ mice (Figures 3A–D). Overall, 15.2 ± 0.2% of Fluorogold-labelled LDPNs were GlyT2 positive (GlyT2-LDPN) and 10.4 ± 0.3% were GAD67 positive (GAD67-LDPN). The vast majority of GlyT2- and GAD67-LDPNs were located either ipsilateral to the injection site (57.4 ± 6.9% and 55.0 ± 12.0%, respectively) or in the midline of the spinal cord (30.8 ± 7.0% and 31.2 ± 4.4%, respectively; Figures 3Ei,Fi). Only a small proportion of inhibitory LDPNs were found contralateral to the lumbar injection site (11.9 ± 4.5% and 13.8 ± 7.6% for GlyT2- and GAD67-LDPNs respectively). Thus, the proportion of ipsilateral inhibitory LDPNs is significantly higher than contralateral inhibitory LDPNs (*p* < 0.01 for GlyT2-LDPNs, and *p* < 0.05 for GAD67-LDPNs; Figures 3Ei,Fi). GlyT2-LDPNs were mostly located within lamina VII (20.4 ± 5.7%) and VIII (26.6 ± 5.1%) of the ipsilateral grey matter and the midline of the spinal cord (lamina X and medial lamina IV). GAD67-LDPNs were predominantly located in the ipsilateral LSN/LCN (38.4 ± 6.2%), and midline of the spinal cord (Figures 3Eii,Fii).

### Developmental Genetics of LDPNs

**Figure 4:**
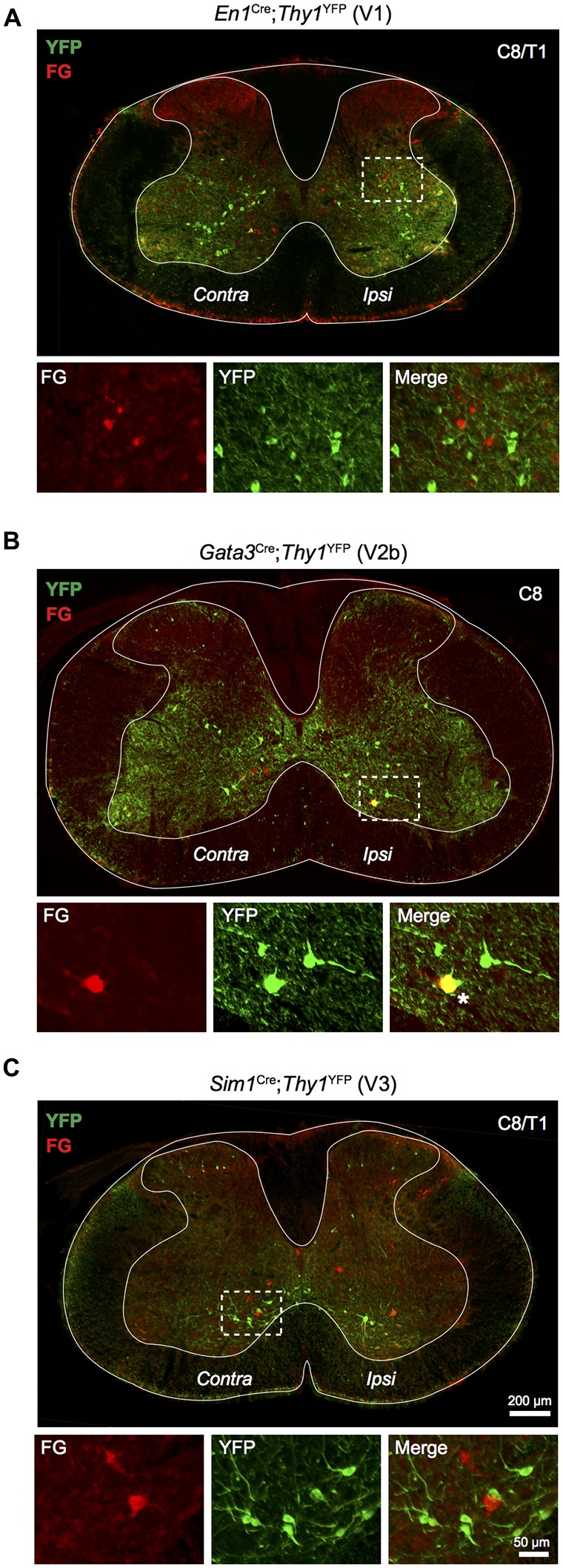
**Relationship of LDPNs with developmentally defined interneuron populations.** Low power images show the distribution of Fluorogold and YFP labelling in cervico-thoracic segments, with high power insets of indicated areas (dashed boxes). **(A)** YFP labelled V1 interneurons (En1 positive; green) rarely colocalised with Fluorogold labelled LDPNs (red). **(B)** V2b interneurons (Gata3 positive; green) formed a small population of ipsilateral lamina VII/VIII LDPNs (red). Asterisk marks an example of Gata3 expression in an LDPN, lower panels. **(C)** V3 interneurons (Sim1 positive; green) rarely colocalised with Fluorogold labelled LDPNs (red).

The developmental origin of LDPNs was assessed using *En1*^Cre^;*Thy1*^floxstop−YFP^, *Gata3*^Cre^;*Thy1*^floxstop−YFP^, and *Sim1*^Cre^;*Thy1*^floxstop−YFP^ mice, which express YFP in V1, V2b and V3 interneurons, respectively. These three ventrally-born interneuron populations were investigated because of the high prevalence of LDPNs in the intermediate and ventral regions of the spinal cord (Figure 2). Additionally, V1, V2b and V3 interneurons perform critical roles in locomotor function and coordination (Zhang et al., 2008, 2014). Surprisingly, LDPNs only exhibited notable colocalisation with V2b interneurons with 5.3 ± 2.4% of LDPNs overlapping with Gata3-positive interneurons (Figure 4B). Only 0.9 ± 0.4% and 0.9 ± 0.6% of LDPNs colocalised with V1 and V3 interneurons (Figures 4A,C). Of the V2b-LDPNs, 90.0 ± 6.3% were located ipsilateral to the lumbar injection site, within laminae VII and VIII. Although V2b-LDPNs only constituted a small fraction of the total number of LDPNs, V2b-LDPNs account for approximately 22.2 ± 8.3% of all ipsilateral lamina VII and VIII LDPNs.

### LDPN Morphology and Sources of Synaptic Inputs

**Figure 5:**
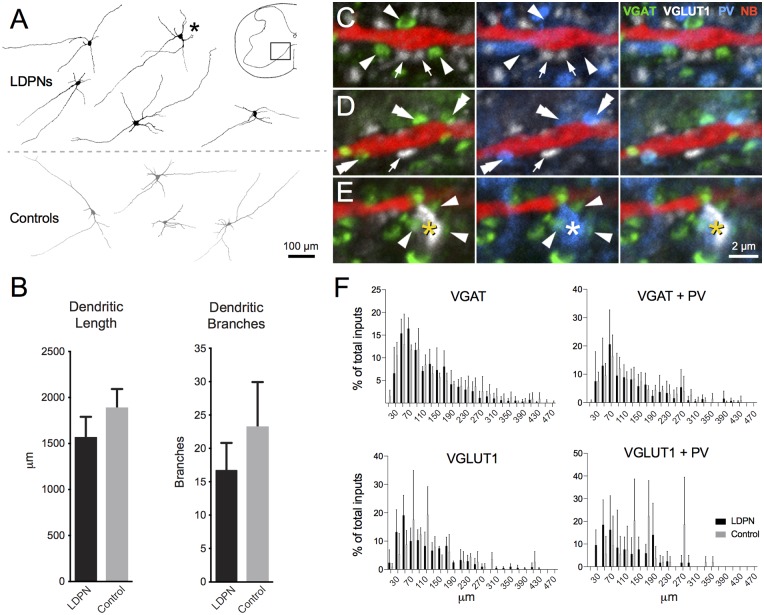
**Morphology of LDPN neurons and putative synaptic inputs. (A)** The morphology of LDPN (black) and control (grey) neurons analysed in this study. These neurons had elongated and sparsely-branching dendritic arbours, orientated primarily in the medio-lateral plane. Asterisk indicates cell from which examples of four classes of neurochemically-defined boutons are illustrated in high power insets **(C–E)**. **(B)** No difference was identified in total dendritic length or number of dendritic branches between LDPN and control neurons. **(C)** Examples of VGAT (green; arrowheads) and VGLUT1 (white; arrows) expressing terminals in close proximity to a Neurobiotin (NB) filled dendrite (red). **(D)** Examples of dual-labelled terminals (double arrowheads) containing both VGAT (green) and parvalbumin (PV; blue). **(E)** Example of a dual-labelled VGLUT1 (white; asterisk) and PV (blue; asterisk) expressing terminal. These terminals were surrounded by VGAT-expressing boutons (green; arrowheads) likely to represent P-boutons. **(F)** Sholl analysis (20 μm bins) of four input types onto LPDN (black) and control (grey) neurons. Column graphs show the proportion of boutons from each neurochemically-defined group that contact dendrites of LDPNs at various distances from their cell body. Data are presented as group means ± SD.

NB-labelled LDPNs located in contralateral lamina VII and VIII were analysed to determine dendritic morphology and the relative frequency of contacts from four neurochemically-defined inputs (*n* = 5). LDPNs were compared with control neurons (i.e., non-fluorescent, unidentified neurons) from the same region (*n* = 4). All NB-filled neurons were from wild-type mice (C57BL6) and located within spinal segments C5 to T1. LDPN and control neurons had relatively simple, radiating dendritic arbours that extended throughout the intermediate zone and ventral horn (Figure 5A). The dendritic arbour of both cell types was generally restricted to less than 200 μm in the rostrocaudal axis. Specifically, the total dendrite length for LDPNs and control neurons ranged from 1106 to 2002 and 1409–2386 μm, respectively, with average dendritic length being similar between groups (1563 ± 262 vs. 1885 ± 239 μm; *p* = 0.3). The number of dendritic branch points in LDPNs and control neurons ranged from 7 to 25 and 12–42, respectively, and these values were also similar for each population (16.8 ± 4.8 vs. 23.3 ± 7.8; *p* = 0.4; Figure 5B).

To examine potential sources of synaptic input onto LDPNs, immunolabelling was performed for several types of inhibitory and excitatory terminal fibres that closely apposed NB-filled neurons. On average, LDPNs received 316.4 ± 27.8 VGAT-positive inputs (Figure 5C). A subset of these inhibitory inputs (90.8 ± 13.5) also expressed PV, suggesting LDPNs receive inputs from Ia inhibitory interneurons (Alvarez et al., 2005; Figure 5D). NB-filled neurons also received contacts from VGLUT1-positive terminals (77.8 ± 15.0), a marker of corticospinal or myelinated-afferent terminals (Todd et al., 2003; Du Beau et al., 2012; Figures 5C,D). Some VGLUT1 terminals (10.0 ± 2.8) were also PV positive, implying that LDPNs receive inputs from proprioceptive afferents (Zhang et al., 1990; Clowry et al., 1997; Figure 5E). These contacts were surrounded by clusters of VGAT-positive terminals, which is characteristic of P-bouton inputs on group Ia muscle afferent terminals (Conradi, 1969; Mackie et al., 2003; Hughes et al., 2005; Figure 5E). Sholl analysis revealed the majority of contacts on LDPNs were located within 200 μm of the cell bodies (Figure 5F).

## Discussion

This study characterised the cell body location, inhibitory neurotransmitter profile, developmental genetics, morphology and likely synaptic inputs of LDPNs in mice. LDPNs were found both ipsilateral and contralateral to the lumbar injection site in approximately even numbers but were localised to different laminae on each side. Ipsilaterally projecting LDPNs were spread diffusely throughout the deep dorsal horn, intermediate zone and ventral horn, with a dense population in the LSN/LCN. In contrast, contralaterally projecting LDPNs are concentrated within the ventromedial quadrant of the ventral horn. Another population was also identified in the midline, located in lamina IV and the dorsal part of lamina X. Inhibitory LDPNs, identified by co-localisation of Fluorogold and GFP in *GlyT2*^GFP^ and *GAD67*^GFP^ mice were found almost exclusively ipsilateral to the lumbar injection site. LDPNs rarely colocalised with developmental neuron classes V1, V2b or V3, however, a discrete population of ipsilateral LDPNs located between laminae VII and VIII are derived from V2b interneurons. Our data on NB-filled, contralaterally projecting LDPNs located in ventromedial spinal cord identified radial dendritic arbours restricted largely to the mediolateral plane. This was similar to the dendritic morphology of the NB-filled comparison group from the same region (i.e., non-DiI labelled). Immunohistochemical labelling of excitatory and inhibitory terminals in close apposition to this population of LDPNs showed a diverse range of convergent inputs including putative premotor inhibitory interneurons, corticospinal fibres and proprioceptive afferents.

### LDPN Cell Body Locations

The tight clustering of LDPN cell bodies contralateral to the injection site suggests this subpopulation forms a more discrete functional group compared to the diffusely distributed ipsilateral LDPNs. As yet, the function of these contralateral LDPNs is unknown. However, their location in the ventromedial grey matter of lamina VII and VIII means they are well positioned to integrate signals from premotor interneurons that make up the cervical locomotor central pattern generator (CPG; Kjaerulff and Kiehn, 1996; Kiehn and Butt, 2003). Furthermore, because of their direct projections to the lumbar spinal cord, contralateral LDPNs could be a conduit for signalling between cervical and lumbar CPGs. In support of this premise, acute inactivation of the comparable long *ascending* propriospinal neurons (LAPNs) inhibits conventional interlimb coordination in mice (Pocratsky et al., 2014). Ipsilateral LDPNs are also found in the ventral grey matter, but also throughout the intermediate zone and deep dorsal horn. This suggests that as well as linking motor output in fore- and hindlimb circuits, ipsilateral LDPNs transmit sensory signals such as nociceptive, mechanoreceptive and proprioceptive inputs to the lumbar cord (Heise and Kayalioglu, 2009). It is important to note that a portion of the ipsilateral LDPNs labelled in this study are also likely to have commissural axons that cross the midline prior to reaching the L2 spinal segment (Reed et al., 2006).

The segmental and laminar distribution of mouse LDPNs mapped in this study closely mirrors that observed in the cat monkey, rat, and rabbit (Matsushita and Ueyama, 1973; Burton and Loewy, 1976; Molenaar and Kuypers, 1978; Skinner et al., 1979; Menetrey et al., 1985; Conta and Stelzner, 2004; Reed et al., 2006). The only difference we found was the existence of a more dense population of LDPNs in the lateral spinal nucleus and lateral cervical nucleus (LSN/LCN) than that reported for other species (Burton and Loewy, 1976; Molenaar and Kuypers, 1978; Menetrey et al., 1985; Brockett et al., 2013). The LSN has projections to multiple targets throughout the spinal cord, brainstem, midbrain, diencephalon and striatum (Heise and Kayalioglu, 2009), whereas LCN neurons provide ascending projections to supraspinal structures, including the periaqueductal grey (Mouton et al., 2004) and thalamus (Craig and Burton, 1979). Neurons in the LSN and LCN respond to noxious stimuli from somatic, articular and visceral sources (Kajander and Giesler, 1987; Menetrey et al., 1989). Consequently, these LDPNs may form a nociceptive pathway directly connecting the cervical and lumbar cord. Functionally, these direct LDPN connections could be utilised to rapidly engage hindlimb musculature in order to avoid destabilisation during forelimb withdrawal, or to prepare for avoidance/escape behaviour.

### Inhibitory LDPNs

A major finding of this study is that the vast majority of inhibitory LDPNs were located ipsilateral to the lumbar injection site. Despite approximately half of the overall LDPN population projecting contralaterally, only 12%–14% of inhibitory LDPNs exhibited contralateral projections (compare Figure 3 with Figure 2). This difference implies anatomical specialisation based on neurotransmitter phenotype. Further studies assessing the lumbar circuits targeted by these inhibitory LDPN projections are required to understand the functional significance of this population.

The low percentage of inhibitory LDPNs labelled in our study (15.2% GlyT2 positive, and 10.3% GAD67 positive), which is further compounded by the well-established co-expression of glycine and GABA (Todd and Spike, 1993; Jonas et al., 1998), suggests that the majority of LDPNs are excitatory by exclusion. This is supported by the dominance of excitatory projections (85%) in an analogous population of long ascending propriospinal neurons (LAPNs) that link segments L1 to C7/C8 (Brockett et al., 2013). In contrast, a population of premotor LDPNs and descending thoracic PNs that directly innervate tibialis anterior motoneurons contain roughly *equal* numbers of excitatory and inhibitory interneurons (Ni et al., 2014). This may reflect differences between premotor LDPNs and the broader LDPN population examined in this study.

### LDPN Developmental Genetics

We found that a subset of ipsilaterally projecting LDPNs in lamina VII and VIII colocalised with the developmentally defined V2b interneuron population. V2b interneurons develop from the p2 progenitor domain in the ventral spinal cord alongside V2a (Chx10 positive) and V2c (Sox1 positive) interneurons (Karunaratne et al., 2002; Panayi et al., 2010). V2b neurons are inhibitory (GABA/glycine positive) and have ipsilateral projections that extend throughout the ventral horn to innervate both premotor- and motoneurons (Lundfald et al., 2007; Joshi et al., 2009; Zhang et al., 2014). While the overall function of V2b interneurons remains unknown (Francius et al., 2014), recent work has demonstrated that a portion of V2b interneurons develop into Ia interneurons that provide reciprocal inhibition to hindlimb motoneurons (Zhang et al., 2014). In combination with V1 (En1 positive) derived Ia interneurons, they permit flexor-extensor alternation for appropriate motor control of the hindlimbs to form a critical component of the locomotor CPG (Zhang et al., 2014). Our data shows that V2b interneurons also provide direct, ipsilateral connections between the cervical and lumbar enlargements. Recently, the developmentally related excitatory V2a interneurons were shown to form a subset of cervical PNs that project to both forelimb motoneurons and the lateral reticular nucleus. These cervical V2a PNs comprise a critical sensorimotor relay network necessary for skilled reaching behaviour (Azim et al., 2014). Additionally, V2a interneurons form a group of descending thoracic premotor PNs that synapse onto tibialis anterior motoneurons in the lumbar spinal cord (Ni et al., 2014).

Our focus on ventrally derived interneurons arises from well-established roles in effective/smooth quadrupedal locomotion and coordination of movement in all body segments. It is, however, important to note that LDPNs were also identified within the dorsal horn and future work would benefit from investigating potential LDPN populations derived from dorsal progenitor zones (dI0-6; Gross et al., 2002; Gosgnach, 2011). A particularly interesting candidate is the inhibitory dI6 (Dmrt3/WT1 positive) interneuron population, which is critical for establishing normal locomotor patterns between the fore- and hindlimbs to achieve a restricted range of gaits (Andersson et al., 2012; Vallstedt and Kullander, 2013). It is plausible that dI6 interneurons accomplish this through direct projections between cervical and lumbar locomotor CPGs in combination with their established local ipsi- and contralateral projections (Andersson et al., 2012). Future experiments will be required to evaluate the likelihood of a dI6 or other dorsally-born interneurons contributing to the LDPN network.

### LDPN Morphology and Synaptic Inputs

NB-filled, contralaterally projecting LDPNs located in the ventromedial spinal cord exhibited radiating dendritic arbours that branched profusely in the mediolateral plane. Such dendritic architecture is typical of lamina VII and VIII neurons (Schoenen and Faull, 2004). A comparison with control neurons in the same location did not reveal any significant differences in dendritic morphology, suggesting LDPNs do not have specific morphological properties, apart from their long descending axons. Similar morphological profiles have been described in descending commissural PNs located in the intermediate zone and ventral horn of the thoracic spinal cord (Saywell et al., 2011).

Immunohistochemical labelling revealed that contralateral, ventromedial LDPNs receive putative synaptic inputs from both inhibitory and excitatory pathways. These include inputs from segmental inhibitory interneurons (VGAT), inhibitory premotor interneurons such as Renshaw cells and group Ia inhibitory interneurons (VGAT/PV), excitatory corticospinal or myelinated afferent (VGLUT1) and proprioceptive afferent input from the central terminals of group Ia muscle afferents (VGLUT1/PV). While we are unable to confirm the origin of the various axon terminals apposed to the LDPN or unlabelled comparison group in our study, recent work has confirmed the presence of corticospinal, descending 5-HT and intraspinal inputs onto LDPNs (Ni et al., 2014). Electrophysiological data also shows that several other supraspinal motor centres located in the cortex, cerebellum and brainstem, as well as primary afferents provide monosynaptic input to LDPNs (Brink et al., 1985; Alstermark et al., 1987a,b,c).

Collectively, these observations position the LDPN population as an integrating node for convergent supraspinal, intraspinal and sensory afferent inputs. A similar organisation was recently described for the so-called motor synergy encoder (MSE) spinal neurons located in lamina V (Levine et al., 2014). MSE neurons receive convergent corticospinal and sensory afferent input, and in-turn drive coordinated motor output through activation of specific motoneuron networks. As LDPNs have direct connections with the lumbar cord, they are ideally placed to update and modulate lumbar/hindlimb spinal circuits in response to sensory feedback from forelimb movement, supraspinal command signals, and cervical intraspinal circuitry.

## Conclusion

This study serves to expand our knowledge on the cell body location, inhibitory neurotransmitter profile, developmental genetics, morphology and synaptic inputs of LDPNs throughout the cervical and upper thoracic spinal cord in mice. Further investigation of these interneurons will provide an important step forward in our understanding of longitudinal pathways within the spinal cord and how they contribute to motor control, sensory integration and recovery from SCI.

## Author Contributions

JRF, BAG and DIH: experimental design; JRF, TV, DKH, KAB and VLC performed experiments; JRF, VLC and KAB: data analysis; MDW: development of critical reagents; BAG, RJC, MDG and DIH: experimental supervision; JRF, BAG, RJC and DH: manuscript draft; JRF, MDG, BAG, RJC, DIH, MW, TV, VLC and KAB: final manuscript preparation and approval.

## Funding

This work was supported by the National Health and Medial Research Council of Australia (Grants 628765, 631000 and 1043933 to BAG and RJC), the National Institutes of Health (NS090919 to MDG), the Hunter Medical Research Institute (Grants HMRI11-51; Jennie Thomas Exchange Scholarship to JRF) and the Biotechnology and Biological Sciences Research Council (BBSRC; grant BB/J000620/1 to DIH).

## Conflict of Interest Statement

The authors declare that the research was conducted in the absence of any commercial or financial relationships that could be construed as a potential conflict of interest.
